# Multiple system atrophy pathology is associated with primary Sjögren’s syndrome

**DOI:** 10.1172/jci.insight.138619

**Published:** 2020-08-06

**Authors:** Kyle S. Conway, Sandra Camelo-Piragua, Amanda Fisher-Hubbard, William R. Perry, Vikram G. Shakkottai, Sriram Venneti

**Affiliations:** 1 Department of Pathology, Michigan Medicine, University of Michigan, Ann Arbor, Michigan, USA.; 2 Department of Pathology, Homer Stryker M.D. School of Medicine, Western Michigan University, Kalamazoo, Michigan, USA.; 3 Department of Neurology, Department of Molecular and Integrative Physiology Michigan Medicine, University of Michigan, Ann Arbor, Michigan, USA.

**Keywords:** Neuroscience, Autoimmune diseases, Movement disorders, Neurodegeneration

## Abstract

**BACKGROUND:**

Our objective was to investigate whether primary Sjögren’s syndrome (pSS) is associated with multiple system atrophy (MSA).

**METHODS:**

We performed a retrospective cohort study assessing (a) rates of MSA in a cohort of patients with pSS and (b) rates of pSS in a cohort of patients with MSA. These data were compared with rates in respective control groups. We additionally reviewed the neuropathologic findings in 2 patients with pSS, cerebellar degeneration, parkinsonism, and autonomic dysfunction.

**RESULTS:**

Our cohort of 308 patients with pSS had a greater incidence of MSA compared with 4 large population-based studies and had a significantly higher prevalence of at least probable MSA (1% vs. 0%, *P* = 0.02) compared with 776 patients in a control cohort of patients with other autoimmune disorders. Our cohort of 26 autopsy-proven patients with MSA had a significantly higher prevalence of pSS compared with a cohort of 115 patients with other autopsy-proven neurodegenerative disorders (8% vs. 0%, *P* = 0.03). The 2 patients we described with pSS and progressive neurodegenerative disease showed classic MSA pathology at autopsy.

**CONCLUSION:**

Our findings provide evidence for an association between MSA and pSS that is specific to both pSS, among autoimmune disorders, and MSA, among neurodegenerative disorders. The 2 cases we describe of autopsy-proven MSA support that MSA pathology can explain neurologic disease in a subset of patients with pSS. These findings together support the hypothesis that systemic autoimmune disease plays a role in neurodegeneration.

**FUNDING:**

The Michigan Brain Bank is supported in part through NIH grant P30AG053760.

## Abstract 2

This single-center retrospective cohort study shows an association between primary Sjogren’s syndrome and multiple system atrophy pathology.

## Introduction

There is growing evidence for a causal relationship between neurodegeneration and neuroinflammation. Some neurodegenerative disorders, including variants of frontotemporal lobar degeneration (FTLD) and amyotrophic lateral sclerosis (ALS), have been associated with various systemic autoimmune disorders (1, 2). For example, one large study showed higher rates of preexisting autoimmune disease in patients with ALS, including multiple sclerosis, polymyositis, and Sjögren’s syndrome, when compared with expected population-based numbers. In a population-based case-control study, use of immunosuppressants was associated with lower risk of Parkinson disease, suggesting a causal relationship between neuroinflammation and neurodegeneration (3). However, the extent to which specific autoimmune disorders are associated with distinct neurodegenerative disorders remains relatively unexplored.

Primary Sjögren’s syndrome (pSS) is a chronic autoimmune disorder that affects lacrimal glands and salivary glands, resulting in dryness of the eyes and mouth (4, 5). The annual incidence of pSS is approximately 4–5 cases per 100,000 (5). Up to 20% of patients with pSS experience some neurologic symptoms, most commonly peripheral neuropathy, but also central nervous system manifestations (6).

Some features of pSS-associated neurologic involvement, including cerebellar symptoms and autonomic dysfunction, overlap with clinical features of multiple system atrophy (MSA). MSA is a rare progressive neurodegenerative disease that causes autonomic dysfunction, parkinsonism, and cerebellar dysfunction. The annual incidence is between 0.2 and 0.6 cases per 100,000 (7–11). Pathologically, MSA is characterized by atrophy of the striatum, basis pontis, inferior olivary nucleus, and/or cerebellum, accompanied by α-synuclein immunoreactive glial cytoplasmic inclusions in oligodendroglia (12, 13). As with many other neurodegenerative disorders, MSA pathology is accompanied by significant neuroinflammation, particularly in the form of activated microglia (14, 15). Moreover, there is evidence that neuroinflammation in MSA both causes and responds to abnormal protein aggregation (16).

Because a subset of patients with pSS experience CNS symptoms that overlap with MSA, and because MSA has a strong relationship with neuroinflammation, we hypothesized that MSA pathology may be associated with pSS in some subset of patients with neurologic involvement. Our objective was to evaluate this hypothesis using a retrospective study to determine (a) whether there is a higher incidence of MSA in a cohort of patients with pSS compared with that in general population-based studies; (b) whether MSA is more prevalent in a cohort of patients with pSS compared with patients with other autoimmune diseases; and (c) whether pSS is more prevalent in a cohort of patients with MSA compared with patients with other autoimmune diseases. Additionally, we describe MSA pathology identified at autopsy in 2 patients with suspected neurologic involvement of pSS.

## Results

### Patients with pSS have a higher incidence of MSA compared with participants in general population-based studies

**Figure 1:**
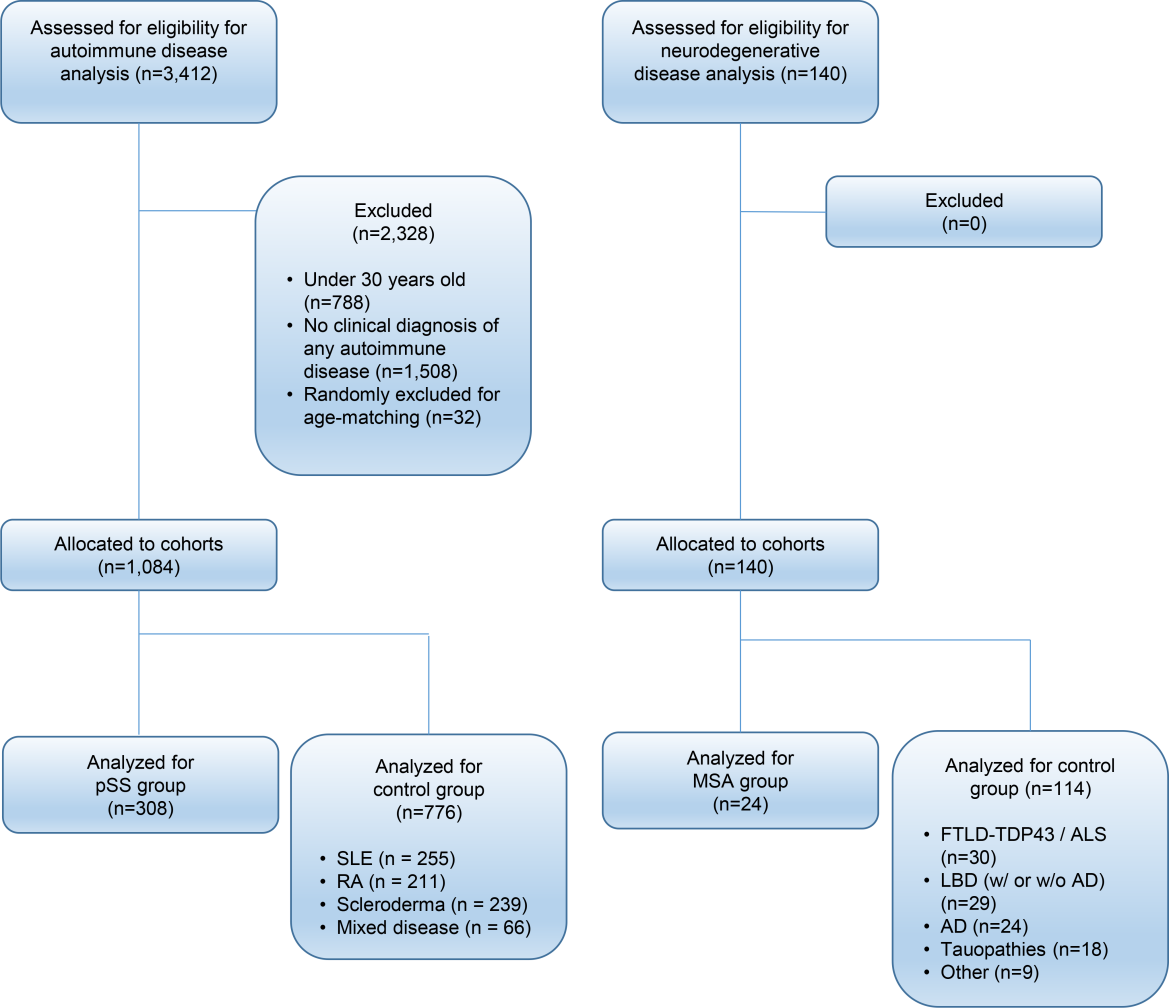
CONSORT diagram of case selection and allocation. pSS, primary Sjögren’s syndrome; SLE, systemic lupus erythematosus; RA, rheumatoid arthritis; MSA, multiple system atrophy; FTLD-TDP43, frontotemporal lobar degeneration–TDP43; ALS, amyotrophic lateral sclerosis; LBD, Lewy body disease; AD, Alzheimer’s disease.

To determine whether there is an association between pSS and MSA, we examined prevalence and incidence of MSA in 5 independent, nonoverlapping data sets. The first group was our pSS patient data set obtained from the University of Michigan; it consisted of a total of 308 patients who met pSS inclusion criteria (Figure 1). Their mean age was 57.8 years, 250 participants (81%) were women, and 256 (83%) were White, 36 (12%) were Black, and 16 (5%) were other/unknown races. All carried a clinical diagnosis of Sjögren’s syndrome, 145 participants (47%) had positive anti-Ro/SSA antibodies, and 68 (22%) had positive anti-La/SSB antibodies.

Among patients in the pSS cohort, all experienced symptoms of ocular or oral dryness and all had a positive lip biopsy. Based on available patient data, 212 patients (69%) met the American College of Rheumatology/European League Against Rheumatism (ACR/EULAR) criteria for the diagnosis of pSS: 145 (47%) by virtue of a positive lip biopsy and positive anti-Ro/SSA antibodies, 63 (20%) by virtue of a positive lip biopsy and positive Schirmer’s test, and 4 (1%) by virtue of a positive lip biopsy and other objective criteria (ocular staining score or salivary flow rate). The remaining 96 patients not meeting ACR/EULAR criteria were diagnosed on the basis of various objective clinical factors, including positive anti-La/SSB antibodies in the absence of positive anti-Ro/SSA antibodies (24 patients), other objective serologic evidence of systemic autoimmunity (27 patients), additional objective clinical evidence of salivary/ocular dysfunction (26 patients), otherwise unexplained peripheral neuropathy (21 patients), and interstitial lung disease without another well-defined connective tissue disorder (17 patients).

Within the pSS cohort, we identified 3 patients with at least probable MSA: 2 with definite MSA and 1 with probable MSA (Table 1).The annual lifetime incidence rate of MSA in the pSS cohort was 17 cases per 100,000 person-years (95% CI = 4.3–46), based on the presence of 3 cases and a total of 17,803 person-years assessed. In addition to the 3 patients with probable or definite MSA, we identified 1 patient with a cerebellar syndrome (ataxia and dysmetria on exam) and autonomic failure (neurogenic bladder requiring catheterization). Based on these findings, this patient met clinical criteria for possible MSA, although other explanations for these findings are possible, and this patient was not included in our overall incidence calculation. Two additional patients showed progressive cerebellar ataxia and cerebellar atrophy/degeneration on imaging but did not otherwise exhibit clinical features of MSA.

In addition to MSA and MSA-associated symptoms, a number of other central nervous system manifestations were observed among patients in the pSS cohort. Five patients experienced radiologically demonstrated transverse myelitis, one of whom had positive serum aquaporin-4 antibodies detected at Mayo Clinic, and five patients showed radiologic evidence of demyelination consistent with multiple sclerosis. The remaining CNS manifestations included 2 patients with aseptic meningitis, 1 with an unexplained adult-onset seizure disorder, and 1 with unexplained chorea. No overlap was noted between patients with MSA and patients with other CNS manifestations.

**Figure 2:**
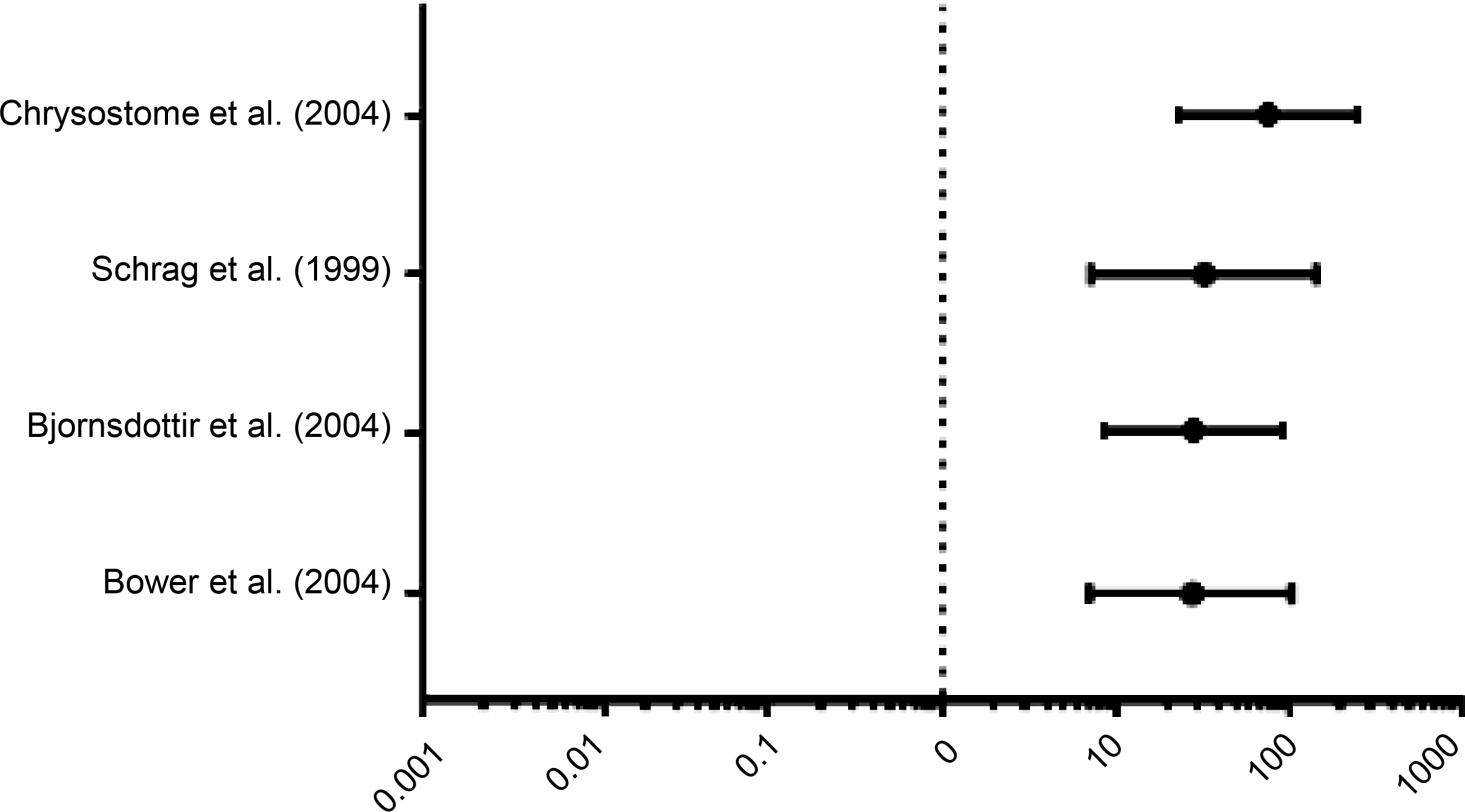
Incidence rate ratios of MSA in the pSS group compared with the general population. Incidence ratios were calculated based on the lifetime annual incidence of MSA in the pSS group compared with the incidence calculated (or estimated) from each study. Error bars represent 95% CIs. The 95% CIs for each incidence rate ratio were calculated manually using the logarithms of the incidence rate ratios.

In addition to our pSS cohort, we examined 4 separate independent studies describing the incidence or prevalence of MSA in the general population. A study by Bower et al. in Olmsted county, Minnesota, USA, and by Bjornsdottir et al. in Iceland, reported incidence data (9, 10). The other two studies, by Schrag et al. in London, United Kingdom, and by Chrysostome et al. in France, recorded prevalence data (8, 11). For the 2 studies with prevalence data, we calculated estimated incidence rates using an 8.5 year median survival. The lifetime annual incidence of MSA in our pSS cohort was significantly higher than the incidence of MSA in all 4 of these studies. The ratios of incidence in our pSS cohort compared with the populations studied were as follows: for Bower et al. (9), 27 (95% CI = 7.2–98.5); for Bjornsdottir et al. (10), 26 (95% CI = 7.7–88.5), for Schrag et al. (8), 32 (95% CI = 7.3–146), and for Chrysostome et al. (11), 77 (95% CI = 23–256) (Table 2 and Figure 2).

### Patients with pSS have a higher prevalence of MSA compared with patients with other autoimmune diseases

To evaluate whether MSA was associated with pSS compared with patients with other autoimmune diseases, we assembled a control cohort of 776 patients. Their mean age was 57.6 years, 607 patients (78%) were women, and 571 (74%) were White, 153 (20%) were Black, and 52 (7%) were other/unknown races. In this control cohort, 79 patients (10%) carried a diagnosis of secondary Sjögren’s syndrome, 148 (19%) had anti-Ro/SSA antibodies, and 51 (7%) had anti-LA/SSB antibodies. Regarding autoimmune disease status, 255 patients (33%) had systemic lupus erythematosus (SLE), 211 (27%) had rheumatoid arthritis (RA), 238 (30%) had scleroderma (diffuse or limited systemic sclerosis), and 66 (9%) had other autoimmune diseases or mixed/overlapping disease. Serologically, among the patients with SLE, 243 (95%) had documented positive antinuclear antibodies (ANAs) and 177 (69%) had anti–double-stranded DNA antibodies; among the patients with RA, 159 (75%) had either a positive rheumatoid factor or anti-CCP antibodies; and among the patients with scleroderma, 123 (51%) had either positive anti–SCL-70, anti–centromere B, or anti-RNA polymerase III antibodies. The prevalence of definite or probable MSA was 0% in this cohort, and 1 patient met clinical criteria for possible MSA.

Compared with the control cohort, the pSS cohort had a significantly higher prevalence of at least possible MSA (1% vs. 0.1%, *P* = 0.03), at least probable MSA (1% vs. 0%, *P* = 0.02), progressive cerebellar syndromes (2% vs. 0%, *P* < 0.01), autonomic failure (3% vs. 1%, *P* < 0.01), and radiologic cerebellar atrophy (2% vs. 0%, *P* < 0.01) (Table 3). The presence of anti-Ro antibodies, regardless of pSS diagnosis, was associated with at least probable MSA (*P* = 0.02) and radiologic cerebellar atrophy (*P* = 0.049). Sjögren’s syndrome, when evaluated independent of primary or secondary status, was associated with at least probable MSA (1% vs. 0%, *P* = 0.045), autonomic failure (3% vs. 1%, *P* < 0.01), a cerebellar syndrome (2% vs. 0%, *P* < 0.01), and radiologic cerebellar atrophy (2% vs. 0%, *P* < 0.01). The sole patient in the control cohort with a cerebellar syndrome and radiological cerebellar atrophy (without other MSA features) notably had secondary Sjögren’s syndrome with RA as a primary diagnosis.

### The increased prevalence of pSS is specific to MSA, among other neurodegenerative disorders

Having established a higher prevalence of MSA in patients with pSS compared with other autoimmune disorders, we next assessed the prevalence of pSS in neurodegenerative disorders. We assembled a retrospective cohort of 140 randomly chosen patients with autopsy-established neurodegenerative disease, including MSA (*n* = 26), frontotemporal lobar degeneration-TDP43/amyotrophic lateral sclerosis spectrum disorders (*n* = 34), Lewy body disease with or without coexisting Alzheimer’s disease pathology (*n* = 29), Alzheimer’s disease (*n* = 24), other tauopathies (*n* = 18), and other diseases or mixed/overlapping pathology (*n* = 9). Their mean age was 72.1 years, 133 patients (95%) were White (patient race was unknown in 2 cases [5%]), and 78 patients (56%) were women.

In this cohort, the association between pSS and MSA mirrored our previous results. The MSA cohort had a significantly higher prevalence of pSS compared with the control neurodegenerative disease cohort (8% vs. 0%, *P* = 0.03). Among all patients, there was a higher prevalence of anti-Ro/SSA antibodies in the MSA cohort (12% vs. 0%, *P* < 0.01). However, testing for ANAs was far more common in the MSA cohort than the other neurodegenerative disease cohort, with 15 patients (58%) with MSA having documented testing for ANAs during their lifetime and only 8 control patients (7%) having documentation of the same testing. This statistical relationship did not remain when restricting the comparison only to those patients who were tested for autoantibodies (*P* = 0.53) (Table 4).

### Neuropathology of pSS-associated MSA

Our analyses in multiple, nonoverlapping cohorts show evidence of an association between pSS and MSA that is specific to both pSS (among autoimmune diseases) and MSA (among neurodegenerative diseases). To define the neuropathology of pSS-associated MSA, we queried our pathology archives. Two patients with pSS and MSA had full autopsy evaluation with a neurodegenerative workup. The clinical and pathologic features of each patient are described below.

### Patient 1

#### Clinical presentation.

The patient was a 68-year-old woman with insidious onset of balance difficulties in the context of prior 8-year alcohol abuse. She had progressive ataxia in spite of minimal alcohol use 2 years following symptom onset. She was diagnosed with idiopathic cerebellar ataxia 5 years following symptom onset, with neuroimaging showing prominent midline cerebellar atrophy. She was noted to have ataxic dysarthria, an axial tremor, and appendicular dysmetria. She did not report orthostatic symptoms, urinary retention, or dream enactment behavior. At her initial visit she was evaluated with a rheumatologic screen. An ANA dilution of 1:160 and a positive SSA antibody level were detected. SSB antibody was negative. Subsequent CSF studies showed an elevated IgG index as well as 3 oligoclonal bands in her spinal fluid, 2 of which were also present in serum. Given the possibility that this could be a neurologic manifestation of pSS, sicca symptoms were identified and a salivary gland biopsy showed lymphocytic infiltration, consistent with a diagnosis of pSS. She was treated with extended-release propranolol for tremor and high-dose oral prednisone and received 2 doses of intravenous cyclophosphamide for pSS-associated cerebellar ataxia. The was no improvement in ataxia following immunosuppression. She developed symptomatic orthostatic hypotension in the context of a hospitalization for cyclophosphamide infusions. She died 6 weeks following the second infusion of cyclophosphamide.

#### Neuropathological findings.

Grossly, there was severe, diffuse cerebellar and pontine atrophy. The substantia nigra and locus coeruleus were mildly hypopigmented. Cut sections showed diffuse, severe loss of cerebellar white matter not confined to the vermis. The mammillary bodies were unremarkable. Microscopically, there was severe Purkinje cell dropout, cerebellar white matter atrophy, and atrophy of the pontine crossing fibers. Staining for α-synuclein showed moderate-to-frequent glial and neuronal cytoplasmic inclusions and focal intranuclear inclusions in the cerebellum (white matter and granular layer), cerebral peduncle, pontine white matter, medulla, putamen, thalamus, and subthalamic nucleus. There was focal endoneurial and perivascular inflammation within cranial nerve V that consisted of CD4^+^ and CD8^+^ T cells (CD3). Tau, β-amyloid, and TDP-43 were negative. No evidence for vasculitis was noted. These neuropathologic findings established a diagnosis of definite MSA.

### Patient 2

#### Clinical presentation.

**Figure 3:**
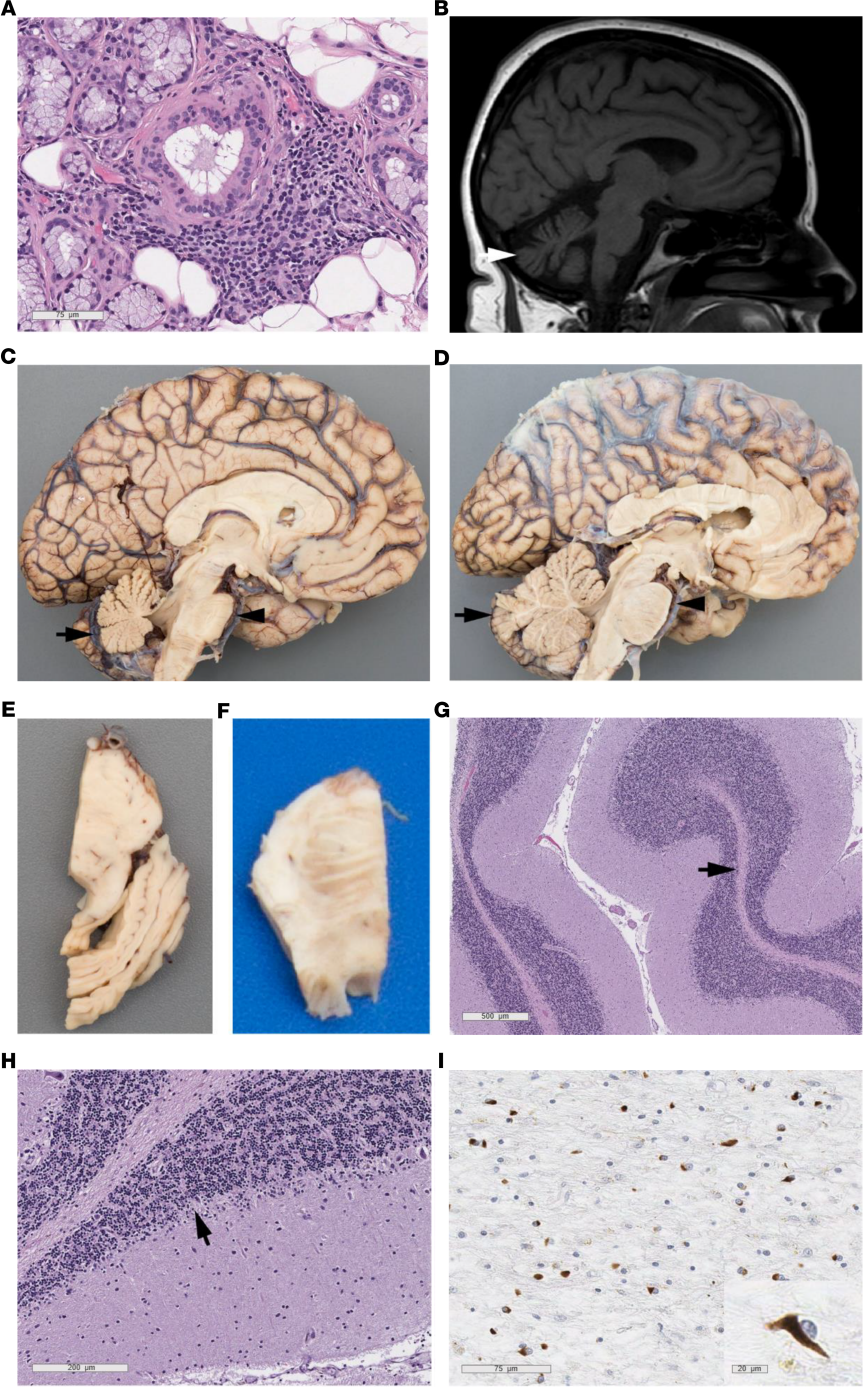
Representative radiologic and pathologic findings from patient 2. Salivary gland biopsy showing lymphocytic sialadenitis (focus score = 2) (**A**). MRI showing cerebellar atrophy (**B**). At autopsy, there was macroscopic evidence of cerebellar atrophy (black arrow) and pontine atrophy (arrow) (**C**), compared with a control autopsy specimen with a normal pons and cerebellum (**D**). Cross section of the pons showing atrophy, loss of smooth outward convexity on the anterior aspect, and flattening of the base (**E**), compared with a normal control pons (**F**). H&E-stained sections of the cerebellum showing atrophy and thinning of the white matter (arrow) (**G**) and Purkinje cell dropout with accompanying Bergmann gliosis (**H**). Numerous glial cytoplasmic inclusions were seen in the cerebellar white matter, predominant with the flame or sickle shape characteristic of MSA (inset, scale bar: 20 μm) (**I**). Scale bar: 75 μm (**A** and **I**); 200 μm (**H**); 500 μm (**G**).

The patient was a 62-year-old woman with plaque psoriasis. She was evaluated initially at age 59 with a 2-year history of dizziness and balance difficulties. She was noted to have cerebellar dysarthria, limb dysmetria, and gait difficulties. Mild cerebellar atrophy was observed on the brain MRI. The patient was found to have a positive ANA and SSA/anti-Ro antibody. A lip biopsy showed chronic sialadenitis (Figure 3A). This, together with her sicca symptoms, confirmed the diagnosis of pSS. She underwent infusions of cyclophosphamide for pSS-associated cerebellar ataxia. She had an improvement in ataxia with improved gait, alternating movements, and dysmetria at a 6 month follow-up visit. She was maintained on mycophenolate. She subsequently, however, had a progressive decline in gait over the next 6 months and underwent treatment again with cyclophosphamide and rituximab. An MRI in at age 61 showed progression of diffuse volume loss in bilateral cerebellar hemispheres, with new midbrain, pons, and medulla atrophy (Figure 3B). She developed symptomatic orthostatic hypotension and parkinsonism. At age 62, she reported choking often, frequent urinary tract infections, and was wheelchair dependent. She was transitioned to hospice care and passed away 2 months subsequently.

#### Neuropathological findings.

Grossly, there was moderate-to-severe atrophy of the basis pontis and severe, diffuse atrophy of the cerebellar white matter (Figure 3, C–E). The substantia nigra and locus coeruleus were hypopigmented. Microscopically, there was severe thinning and gliosis of white matter and diffuse loss of Purkinje cells in the cerebellum (Figure 3, F–H). There was severe atrophy of the pons and middle cerebellar peduncle, loss of pontine neurons, and increased gliosis. A full neurodegenerative evaluation demonstrated α-synuclein^+^ moderate-to-severe glial cell inclusions and synuclein positive threads in the cerebellum, midbrain, pons, basal ganglia, and inferior olivary nucleus (Figure 3I). No evidence for vasculitis was noted. These neuropathologic findings established a diagnosis of definite MSA.

## Discussion

Our data gathered from multiple cohorts show a significant and specific association between pSS and MSA. Three main lines of evidence support this observation: (a) patients with pSS have an increased incidence of MSA compared with the general population; (b) patients with pSS have an increased prevalence of MSA and individual MSA clinical features compared with patients with other autoimmune disorders; and (c) patients with MSA have an increased prevalence of pSS compared with patients with other neurodegenerative disease.

While the etiology and pathologic basis for this association is not known, it is tempting to speculate that pSS may be associated with neuroinflammation in a subset of patients, contributing to the pathogenesis for MSA. Along these lines, T cell–mediated antineuronal inflammation has been described in pSS-related ganglionitis (17). Recent evidence suggests that the oligodendroglial cytoplasmic α-synuclein inclusions in MSA may be the result of α-synuclein released from degenerating neurons (16, 18). CD4^+^ and CD8^+^ T cells, in conjunction with microglial activation, play a role in MSA pathogenesis (19). Thus, MSA might be a secondary result from immune-mediated neuronal injury, although this link remains hypothetical, and the reasons for an association between pSS and MSA are unknown. A spectrum of neuroinflammation has been previously described in patients with pSS, including neuromyelitis optica spectrum disorders (NMOSDs). Although 5 patients in our pSS cohort had evidence of transverse myelitis that was presumed to be pSS related, none of these patients had developed MSA at the time of this study. Whether there is a relationship between the development of NMOSD and MSA in these patients remains speculative.

A corollary of the relationship between pSS and MSA is a role for neuroinflammation in MSA. While there is no obvious neuroinflammation observed on standard H&E sections, MSA tissues do exhibit marked neuroinflammation comprising microgliosis and astrogliosis. This is associated with increased proinflammatory cytokine levels (14, 20, 21). This activation of the innate immune system in the brain can also be associated with a systemic immune dysregulation of a subset of peripheral blood innate immune cells — monocytes (22). The exaggerated innate and adaptive immune responses in pSS (23) overlap with the aberrant activation of innate immunity in MSA.

A subset of patients with pSS develop cerebellar degeneration characterized by ataxia, gait abnormalities, and progressive cerebellar degeneration on MRI. This clinical phenomenon was first described in 1994 by Terao, et al. who reported a patient with pSS-associated cerebellar degeneration, based on radiologic cerebellar atrophy (24). Since then, several other case reports and case series have described similar findings of cerebellar degeneration associated with pSS (25–27). Our findings suggest that MSA pathology may explain cerebellar degeneration in a subset of patients with pSS. In addition to the patients with possible, probable, or definite MSA, 2 other patients in our pSS cohort had progressive cerebellar degeneration without meeting any other MSA diagnostic criteria. To our knowledge no studies have yet reported neuropathologic findings in patients with pSS-associated cerebellar degeneration, and autopsy evaluation was not available for the 2 patients with isolated cerebellar degeneration in our pSS cohort. Therefore, the pathologic significance of pSS-associated cerebellar degeneration is unclear, and several possibilities remain: (a) MSA pathology might be present in all cases of pSS-associated cerebellar degeneration, regardless of whether they meet other MSA diagnostic criteria; (b) neuroinflammation in pSS-associated cerebellar degeneration might represent an early stage of disease that potentially progresses to MSA pathology; or (c) pSS-associated MSA and isolated pSS-associated cerebellar degeneration might represent 2 distinct disorders that arise from pSS.

The primary strength of our study is the availability of 2 large patient cohorts and 2 robust control cohorts, which allow us to establish that this association is specific to both pSS and MSA. Although previous studies have described cases of neurologic involvement in pSS, no previous studies have established an association between pSS and MSA. An additional strength of our study is access to full clinical data and neuropathologic evaluation to extensively characterize the pathology of our 2 patients with pSS-associated cerebellar degeneration.

Our study has several key limitations. Primarily, MSA is a rare disease, and even large population-based cohorts detect only small number of patients. Given the rarity of MSA, the presence of even 3 patients with MSA in our pSS cohort is striking. While we found statistically significant relationships between all groups, these relationships must still be interpreted in light of the small number of overall events. The small number of events additionally limits our ability to assess whether the strongest association is between MSA and pSS itself or between MSA and other pSS-associated factors, such as anti-Ro/SSA antibodies; it also limits our ability to assess whether MSA is associated specifically with pSS or with any Sjögren’s syndrome (primary or secondary), although our data strongly suggest the former. The small number of events also limits our ability to control for factors such as race and sex, which may share an epidemiologic relationship to pSS-associated MSA. Further population-based epidemiologic studies should examine these questions.

A second limitation is that this is a retrospective single-center study in a tertiary referral center. This limitation is reflected in some variations in the study population, such as a somewhat lower-than-expected proportion of female patients in the pSS cohort. It is possible that the rate of MSA at the University of Michigan may be higher than that in the general population, and comparisons to the general population should be interpreted in this light. By including an internal control cohort of patients with autoimmune disease, we have substantially mitigated the possibility that the increased rates of MSA in our pSS cohort is due to institutional variation. Additionally, as a retrospective study, full clinical data are not always available. Because all patients were seen in rheumatology or neurology clinics, we inferred that any history suggestive of a movement disorder would be provided, although a significant limitation remains in the fact that some early case presentations may be missed or not provided to the clinician. To broadly detect patients, we used clinical diagnoses for both the pSS group and the control group, and these disorders are sometimes overdiagnosed. In general, among our control group, the prevalence of characteristic antibodies for each autoimmune disease was relatively comparably to published rates for those entities. Notably, all 3 patients with probable or definite MSA had well-established pSS that met ACR/EULAR criteria. Therefore, our estimated rate of rate of MSA would be significantly higher if we limited our analysis only to those patients who met ACR/EULAR criteria.

Finally, Michigan Medicine is a large, tertiary referral center, and these findings would be expected to reflect those in a general population of similar demographic composition, but the single-center nature of our study remains a limitation on the generalizability of this study.

MSA should remain in the differential diagnosis for patients with pSS with CNS symptoms, particularly those with a progressive cerebellar syndrome or cerebellar degeneration by imaging. Autopsy evaluations of these patients, including a full neurodegenerative workup, is important to better characterize this disease. Finally, broader population-based studies of MSA and pSS are warranted to characterize the relationship between the 2 disorders, and further basic and translational research is warranted to elucidate the nature of their pathologic link.

## Methods

### Identification of pSS cohort and control cohorts.

We retrospectively assembled a cohort of University of Michigan patients with pSS to identify patients with MSA. All patients were patients who were seen in rheumatology and/or neurology clinics at Michigan Medicine, a public academic health system wholly owned by the University of Michigan. Our pSS cohort was established from patients at the University of Michigan from 2009 to 2019 with a positive lip biopsy (focus score ≥1) and a clinical diagnosis of pSS based on documentation in rheumatology clinical notes. Patients were excluded from this cohort if they did not carry a clinical diagnosis of Sjögren’s syndrome or had another autoimmune comorbidity (secondary Sjögren’s syndrome). We reviewed individual charts for factors supporting a diagnosis of pSS and to assess for ACR/EULAR criteria for pSS, but we did not require that patients meet these criteria for inclusion. Because the second consensus criteria statement on the diagnosis of MSA defines the disease as an adult-onset disorder (>30 years), we excluded patients who were under 30 years of age (28).

We calculated the lifetime annual incidence of MSA in this cohort and compared this incidence rate in our pSS cohort to incidence rates in the general population. To do this, we search PubMed for search terms including “multiple system atrophy,” “MSA,” “prevalence,” and “incidence.” We included for comparison all studies of any general population cohort evaluating the prevalence or incidence of MSA. According to the second general consensus criteria, the likelihood of MSA is rated as possible, probable, or definite depending on the constellation of clinical features or the presence of autopsy confirmation (28). For comparison to these general population studies, we a priori chose to conservatively include cases in our pSS cohort as MSA where they met criteria for “probable MSA” or “definite MSA.”

We established a control cohort of patients with other autoimmune diseases. These patients were identified by searching the University of Michigan Department of Pathology laboratory information system for patients with diagnoses of SLE, RA, and diffuse or limited systemic sclerosis. These control patients were randomly selected to be age matched to the pSS cohort based on similar proportions of patients with in 10-year age bins. Because MSA is a rare disease, we chose to maximize statistical power by evaluating all cases readily identifiable by a search of the University of Michigan Department of Pathology records.

For both cohorts, we reviewed patient records for demographic factors, antibody status, autoimmune diagnoses, and the following clinical information pertinent to MSA: (a) clinical diagnoses of definite, probable, or possible MSA, as defined by the second consensus criteria (28); (b) individual clinical MSA criteria, specifically parkinsonism, progressive cerebellar syndromes (excluding cases of documented ischemic stroke), and/or autonomic failure; and (3) radiologic evidence of cerebellar or pontine degeneration. All relevant data were obtained via clinical and laboratory records available in the University of Michigan MiChart system. Clinical criteria were assessed by a neurologist with training in movement disorders, who routinely evaluates patients with MSA.

### Identification of the MSA cohort and control neurodegenerative disease cohort.

We assessed the prevalence of pSS among patients with MSA by reviewing brain-only autopsies in the Michigan Brain Bank. To do so, we established a cohort of all MSA cases over the same 10-year period as the pSS cohort (2009–2019) and a second control cohort of randomly selected brain-only autopsies with other neurodegenerative disorders. Control patients were excluded if there were no clinical notes reviewing the patient’s medical history or other sufficient data to establish the presence/absence of pSS. For both the MSA and neurodegenerative control cohorts, we reviewed clinical records for evidence of a clinical diagnosis of pSS and for evidence of autoantibody status. Because MSA is a rare disease, we similarly chose to maximize statistical power by evaluating the maximum number of patients with MSA and control patients readily identifiable by a search of the University of Michigan Department of Pathology records.

### Neuropathologic review.

For the 2 patients with autopsy-established definite MSA, we reviewed autopsy reports, gross photographs, and prepared slides for each case. Both cases had a full neuropathologic evaluation performed on a formalin-fixed hemibrain. This evaluation included gross evaluation, review of H&E-stained slides prepared from paraffin-embedded tissue, and review of antibody-stained slides prepared from paraffin-embedded tissue. Antibodies used for each case included Tau (AT8 antibody; Thermo Fisher, ENMN1020), β-amyloid (6F/3D antibody; Dako, M0872), TDP-43 (polyclonal antibody; Protein Tech, 10782-2-AP), α-synuclein (LB509 antibody; Thermo Fisher, 180215), GFAP (polyclonal antibody; Dako, Z0334), CD4 (SP35 antibody; Ventana, 790-4423), and CD8 (SP57 antibody; Ventana, 790-4460). All technical components were performed in the University of Michigan Department of Pathology histology and immunohistochemistry laboratories according to standardized protocols.

### Statistics.

For comparisons with general population-based studies, we calculated the lifetime annual incidence of MSA in our pSS cohort as the ratio of the number of MSA cases to the total number of person-years evaluated in our pSS cohort. Although MSA by definition occurs after an age of 30 years, we chose to evaluate lifetime annual incidence as the most conservative measure for comparison with population-based studies. Person-years were determined based upon the last available year for which a patient had a clinical note at the University of Michigan reviewing their medical history and performing a review of systems and/or physical examination that included pertinent negatives or positives relevant to diagnostic criteria for MSA. For studies that provided only prevalence data, we estimated incidence in that population from the prevalence data using a median survival of 8.5 years reported by Bower et al. (9) and adopted by Schrag et al. (8) for their calculations estimating MSA incidence from prevalence data. We then calculated the ratio of the incidence in our pSS cohort to the incidence in each population-based cohort. We manually calculated 95% CIs using the logarithm of the incidence rate ratios.

For comparison of MSA rates in the pSS cohort to rates in the control cohort, we calculated prevalence data based on whether the patient had any diagnosis (probable or definite MSA) at the last available clinical time point. Because MSA is a progressive, incurable disease, and because the pSS cohort and the control autoimmune disease cohort were age matched, these prevalence data were determined to minimize bias comparison between the 2 groups. For comparing rates of pSS between the autopsy MSA cohort and control autopsy neurodegenerative cohort, we calculated prevalence of any pSS diagnosis during lifetime or positive pSS-associated antibodies detected at any point in life. Because Sjögren’s syndrome is a chronic autoimmune disorder, this was similarly determined to minimize bias between the groups. For all categorical variables, we assessed for statistical significance using Fisher’s exact test, and for all continuous variables, we assessed for statistical significance using an independent-samples 2-tailed *t* test using R version 3.61 (2019-07-05). We repeated a similar analysis to compare patients with anti-Ro/SSA antibodies to those without and for patients with any Sjögren’s syndrome (primary or secondary) to those without. *P* values of less than 0.05 were considered significant.

### Study approval.

The University of Michigan Institutional Review Board has reviewed all aspects of data collection for this study and determined that it is exempt and not regulated (human subjects protocol numbers HUM00041576 for autopsy/brain bank data, HUM00155351 for secondary data collection of patients in the pSS cohort, and HUM00167888 for secondary data collection of patients in the control autoimmune disease cohort). Consent for all Michigan Brain Bank cases was obtained from the decedents’ next of kin.

## Author contributions

KSC contributed to study design, data acquisition, data analysis, and writing/revising of the manuscript. SCP contributed to data acquisition and writing/revising of the manuscript. AFH contributed to data acquisition and writing/revising of the manuscript. WRP contributed to data analysis and writing/revising of the manuscript. VGS contributed to study design, data acquisition, data analysis, and writing/revising of the manuscript. SV contributed to study design, data acquisition, data analysis, and writing/revising of the manuscript.

## Supplementary Material

Supplemental data

## Figures and Tables

**Table 4:**
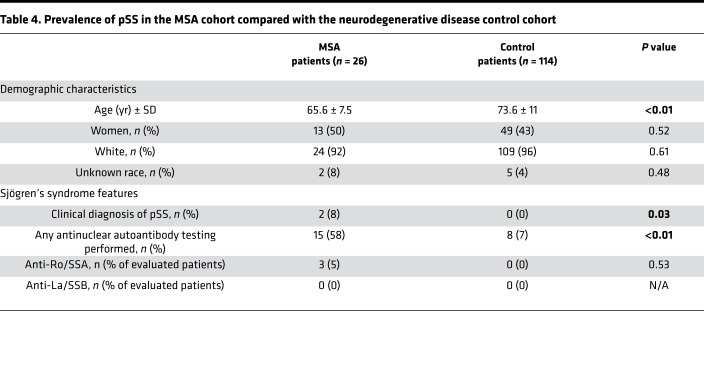
Prevalence of pSS in the MSA cohort compared with the neurodegenerative disease control cohort

**Table 3:**
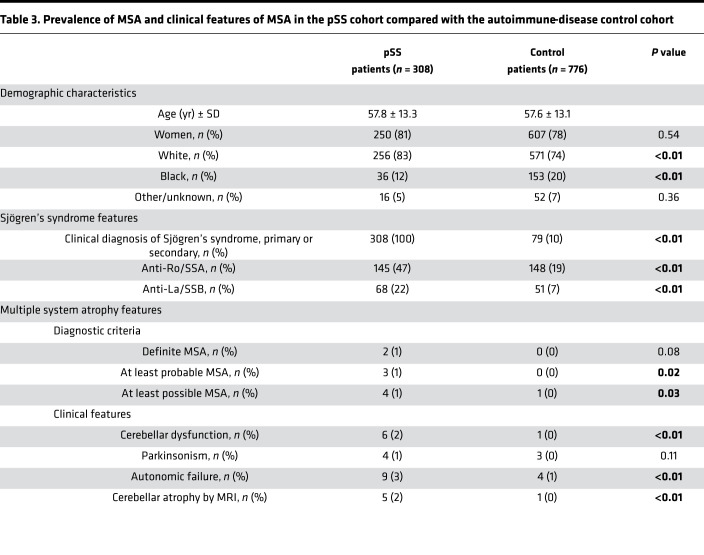
Prevalence of MSA and clinical features of MSA in the pSS cohort compared with the autoimmune-disease control cohort

**Table 2:**
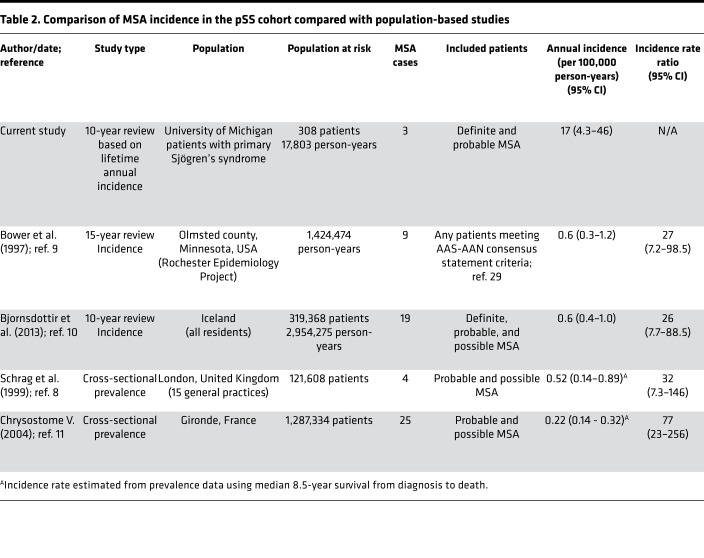
Comparison of MSA incidence in the pSS cohort compared with population-based studies

**Table 1:**
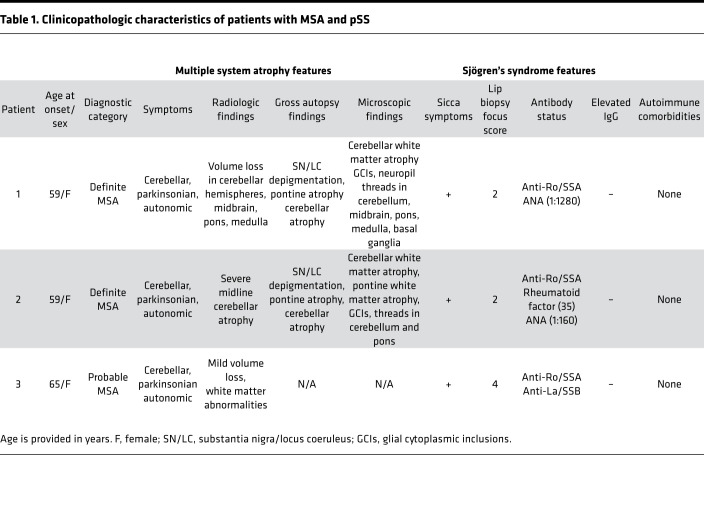
Clinicopathologic characteristics of patients with MSA and pSS

## Version 1 07/09/2020

In-Press Preview

## Version 2 08/06/2020

Electronic publication
